# Self-Organized Micro-Spiral of Single-Walled Carbon Nanotubes

**DOI:** 10.1038/s41598-017-05558-9

**Published:** 2017-07-13

**Authors:** Keisuke Mae, Hidetoshi Toyama, Erika Nawa-Okita, Daigo Yamamoto, Yong-Jun Chen, Kenichi Yoshikawa, Fumiyuki Toshimitsu, Naotoshi Nakashima, Kazunari Matsuda, Akihisa Shioi

**Affiliations:** 1 Department of Chemical Engineering & Materials Science, Doshisha University, Kyoto, 610-0321 Japan; 2 Organization for Research Initiatives and Development, Department of Chemical Engineering & Materials Science, Doshisha University, Kyoto, 610-0321 Japan; 3 Department of Physics, Shaoxing University, Shaoxing, Zhejiang Province 312000 China; 4 Faculty of Life and Medical Sciences, Doshisha University, Kyoto, 610-0394 Japan; 5 Department of Applied Chemistry, Kyushu University, Fukuoka, 819-0395 Japan; 6 International Institute for Carbon-Neutral Energy Research, Kyushu University, Fukuoka, 819-0395 Japan; 7 Institute of Advanced Energy, Kyoto University, Uji, Kyoto 611-0011 Japan

## Abstract

Single-walled carbon nanotubes (SWCNTs) are reported to spontaneously align in a rotational pattern by drying a liquid droplet of toluene containing polyfluorene as a dispersant. By situating a droplet of an SWCNT solution around a glass bead, spiral patterns are generated. The parallel alignment of SWCNTs along one stripe of such a pattern is confirmed using scanning electron microscopy and polarized optical microscopy. The orientation order increases toward the outer edge of a stripe. The stripe width in the pattern is proportional to the solute concentration, and the width and position of the stripes follow geometric sequences. The growth of the rotational pattern is also observed in real time. The process of spiral pattern formation is visualized, indicating the role of the annihilation of counter-traveling accompanied by continuous depinning. The geometric sequences for the stripe width and position are explained by the near-constant traveling speed and solute enrichment at the droplet periphery.

## Introduction

Carbon nanotubes (CNTs) are among the most promising materials for future nanotechnology, exhibiting outstanding optical, electronic, and mechanical properties^1–3^. In most cases, functional materials comprising CNTs contain a mass of CNTs; hence, the alignment of CNTs can be crucial in determining material performance. Thus, various approaches have been proposed to obtain ordered alignment in a desired geometry. A well-known example is the use of chemical vapor deposition (CVD) to obtain a CNT forest growing perpendicular to the solid substrate^4^. The CVD technique also enables parallel alignment of CNTs with respect to a substrate with a photolithographically patterned surface^5^.

In addition to CNT formation from the gas phase, the use of self-organization from or in a liquid containing CNTs may also be powerful in the development of materials design with CNTs^6^. Generally, the fine control of microstructures in liquid processing is difficult, because self-organized patterns are affected by many controlling factors such as included chemicals, chemical concentration, and temperature. However, liquid processing is much simpler than gas-phase operations, and thus the cost performance can be better. Therefore, the study of self-organization from a CNT-containing liquid phase is significant for the technological applications of CNTs. The formation of highly ordered structures has been reported for carbon nanotubes^6–8^; the liquid crystal- and polymer-assisted alignment of CNTs has also been studied^9–11^.

The evaporation of a liquid phase to induce drying patterns of solutes has attracted significant attention from both scientific and technological perspectives^12–21^. This drying method has been investigated to study pattern formation dynamics in physics and used for the design of nano- and microstructured materials. Evaporation patterns can be modified by manipulating the drying procedure. Simple coffee rings form from sessile droplets^12, 13^, while concentric ring or spiral patterns are generated from liquids in confined geometries^14–18, 22, 23^. Other setups, in which vertical plates are soaked in bulk solutions, are also used^9, 24–26^. The solutes dissolved in a droplet also affect the drying pattern^27^. It may be possible to design micro-patterns with novel optical and electronic properties by utilizing the interplay between the properties of the solutes and the formed pattern. In this study, we report the successful generation of specific micro-patterns through drying procedures of liquid droplets containing single-walled CNTs (SWCNTs). To induce the formation of a rotating pattern around a certain fixed position, a droplet of CNT solution is situated surrounding a glass bead. A spiral pattern is easily generated through this drying method. With a perfectly clean and smooth surface, a concentric pattern is generated. CNTs spontaneously align into stripes in this pattern, as confirmed by scanning electron microscopy and polarized optical microscopy. The degree of alignment depends on the position of a CNT in the stripe. The analyzed width and position of each stripe in the pattern indicate the characteristics of self-organized pattern formation under far-from-equilibrium conditions, such as in Liesegang rings. The present study may stimulate further developments in the methodology of pattern formation by CNT alignment.

## Results

### Drying pattern

**Figure 1:**
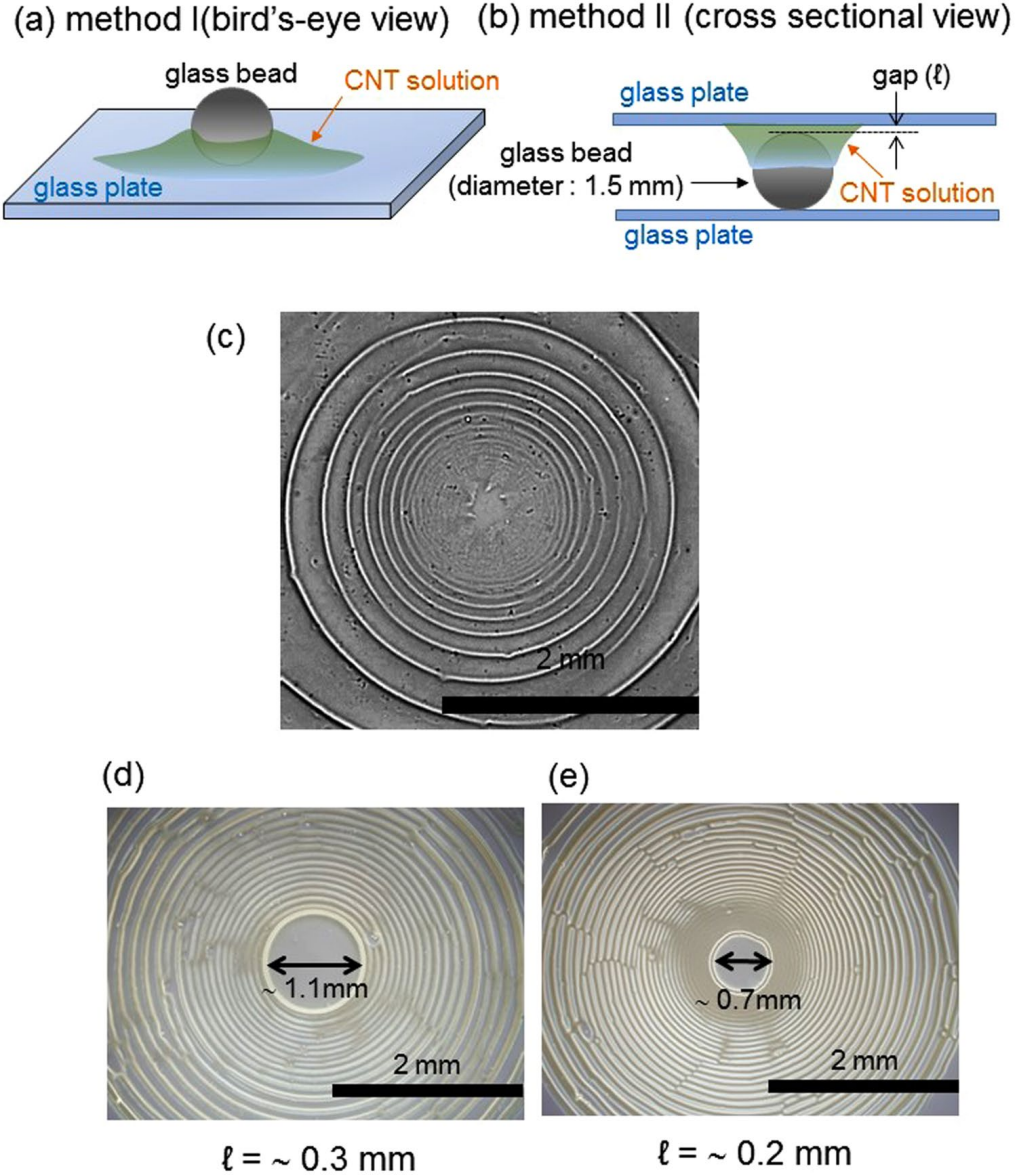
Schematics of two types of experimental setup (**a**,**b**) and drying patterns from the setups; (**c**) Pattern from Fig. 1a setup. (**d**,**e**) Patterns from Fig. 1b setup. The gap between the top of the glass bead and the upper glass plate, shown in Fig. 1b, is of different sizes in (**d**) and (**e**). The concentrations of PFO and SWCNT are 1.0 g/L and 0.33 g/L, respectively. A glass plate is used as the solid surface.

The sample preparation^28^ and drying method^14, 18^ are essentially the same as those reported previously. Figure 1 illustrates the drying method^14, 18^. A drop of the SWCNT solution is placed around a glass bead (Fig. 1a) on a glass plate or between the top of a glass bead and plate (Fig. 1b) and dried in either atmosphere or vacuum at room temperature until the solvent is completely evaporated. The details of experimental procedures are explained in Method section.

Figure 1c shows the “coffee-ring” pattern for the Fig. 1a setup (method I) using phase-difference optical microscopy, indicating the generation of a spiral pattern. Notably, the generation of a spiral as a coffee-ring pattern of fullerenes was previously reported^14, 18^. We have confirmed the appearance of spiral patterns under the same experimental conditions from a PFO solution without SWCNTs (Supporting Figure S1 in the supporting information). These results imply that the generation of the spiral is mainly attributable to the evaporation kinetics of the PFO solution.

**Figure 2:**
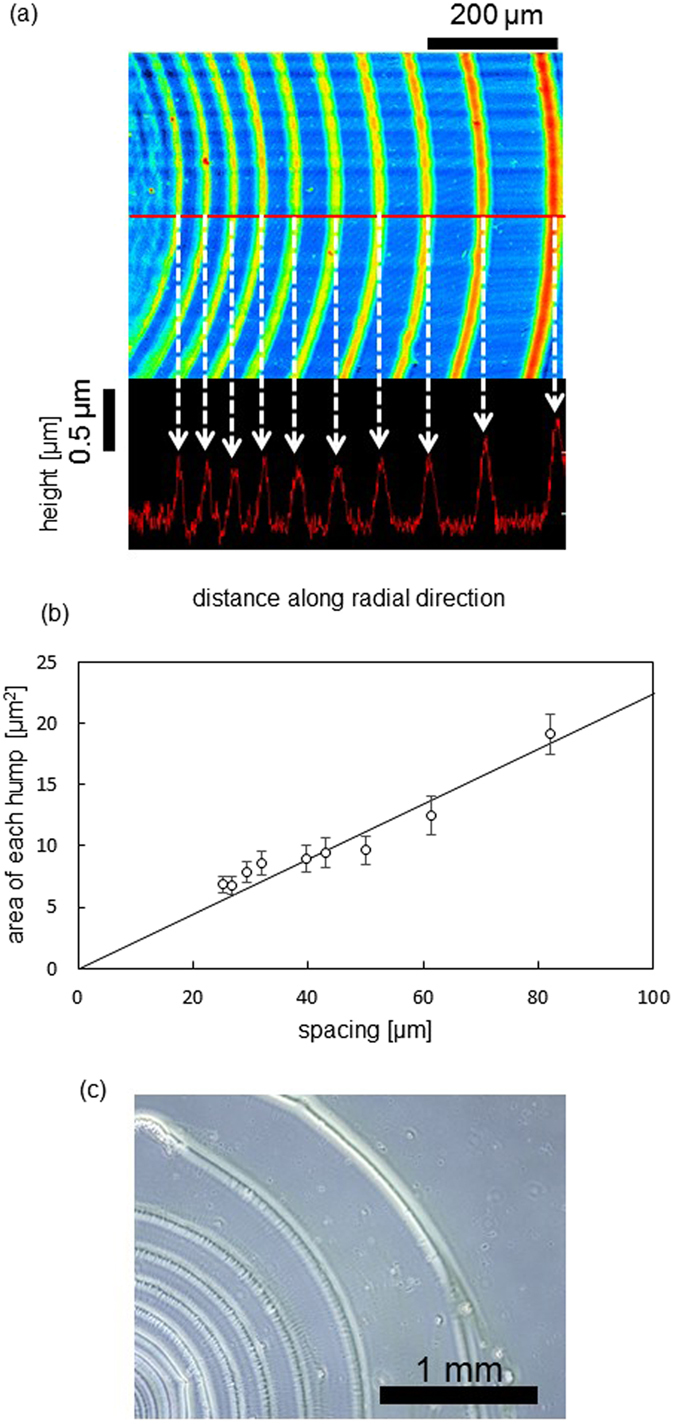
Laser microscopy image with height analysis of the drying pattern under atmosphere (**a**) and the spacing of stripes and the cross-sectional area of each hump (**b**) are shown. (**c**) An optical microscopy image of the drying pattern under vacuum. The concentrations of PFO and SWCNT are 1.0 g/L and 0.33 g/L, respectively. A glass plate is used as the solid surface.

The drying pattern for the Fig. 1b setup (method II) is shown in Fig. 1d and e, where the gap shown in Fig. 1b is approximately 0.3 and 0.2 mm, respectively. A spiral pattern is generated with a spacing approximately 0.25 times less than that obtained on a simple glass plate, as in Fig. 1a. This narrowing of the periodicity is attributed to the difference in the evaporation rate, as well as the difference of the meniscus between the conditions of Fig. 1a and b. The pattern of method I can be more easily analyzed quantitatively, because the dense pattern of method II tends to contain defects such as stripe cutting and dislocations. Thus, method I is mainly used for the present study. Figure 2a shows the laser micrograph of the drying pattern formed by method I. The height distribution along a line is shown. Humps, each corresponding to the cross-section of a stripe, are visible. The height decreases nearer the center of the spiral (toward the left side of the micrograph). Each hump is triangular in shape, exhibiting a long base (~50 μm) and a short height (~0.5 μm). Here, each stripe is defined as the region between the boundaries of the green-colored area, and the spacing of the stripes and the area of each hump are plotted in Fig. 2b. The space inside (left-side of) each hump is taken as the area. The quantities of spacing and area show an approximately proportional relationship. This implies that each hump is formed by the convective transport of solutes in the regions between neighboring stripes. When drying is performed under a vacuum, each stripe contains a narrow periodic pattern along the radial direction, as shown in Fig. 2c. Hereafter, we focus on the regular pattern generated under atmospheric pressure, in which smooth stripes are generated, different from those shown in Fig. 2c.

**Figure 3:**
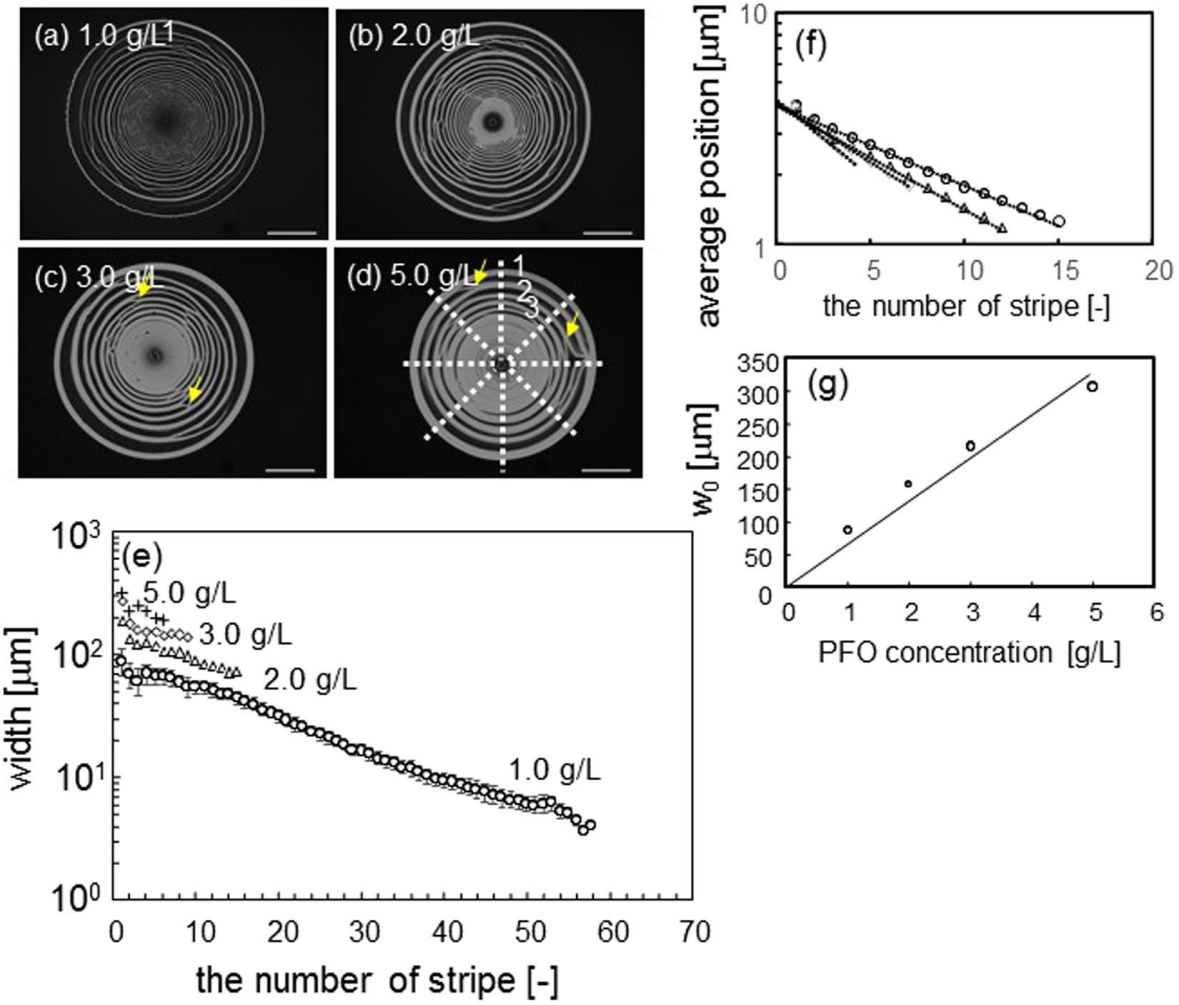
Geometric characteristics of drying pattern. Samples containing PFO only were used. (**a**–**d**) Fluorescence image of PFO. The concentration is shown, and the yellow arrows indicate structural blanching. (**e**) Width of the stripes as a function of stripe number is shown. The stripe number is defined as shown in (**d**). Experiments were performed three times for each of the PFO concentrations. The widths were measured along eight lines, as shown in (**d**), for the three images. Thus, 24 sets of data for the width vs. stripe number were obtained for each PFO concentration. The average and the range were plotted for 1.0 g/L, while only the average was plotted for the other concentrations to allow graphical clarity. (**f**) The distance between the position of the *n*-th stripe and the center is plotted against the stripe number. Each stripe has finite width and the position is defined as the average of the outer and inner periphery locations. (How to determine the width and position is explained in Figure S3) Each key denotes the PFO concentration and is the same as that for (**e**). (**g**) The width extrapolated to *n* = 0 in (**e**) is plotted against the PFO concentration. A glass plate is used as the solid surface.

The coffee-ring pattern is largely dependent on the concentration of PFO, as depicted in Fig. 3a–d. This is expected, because increases in PFO concentration imply increased amounts of solute to form the stripes. Figure 3e shows the width as a function of the stripe number, as counted from the outmost periphery of Fig. 3a–d. The width (*w*_n_) exponentially decreases with increases in the stripe number *n*. The exponent (*α*) of *w*_n_ ∝ exp(−*αn*) is nearly constant, independent of the PFO concentration, at approximately 0.06. This suggests the recurrence relation *w*_n+1_/*w*_n_ = exp(−*α*). Figure 3f shows the distance between the *n*-th stripe and the center of the pattern (*r*_n_). The result indicates *r*_n_ ∝ exp(−*βn*) and *r*_n+1_/*r*_n_ = exp(−*β*). The exponent *β* is dependent on the PFO concentration, expressed as *β* ~ 0.017 [PFO](g/L) + 0.069 (Supporting Figure S2).

The width, extrapolated to *n* = 0 as *w*_0_, is thus evaluated from the exponential function for *w*_n_. Figure 3g shows that *w*_0_ is proportional to the PFO concentration. The results of Fig. 3e and g demonstrate that *w*_n_ at a fixed *n* is also proportional to the PFO concentration. This suggests that the decrease in width accompanying increases in the stripe number is caused by decreases in the PFO concentration during the pattern formation. As the PFO concentration decreases, evaporation speed increases. This may cause decreases in stripe width.

### Pattern formation dynamics: Real-time observation

**Figure 4:**
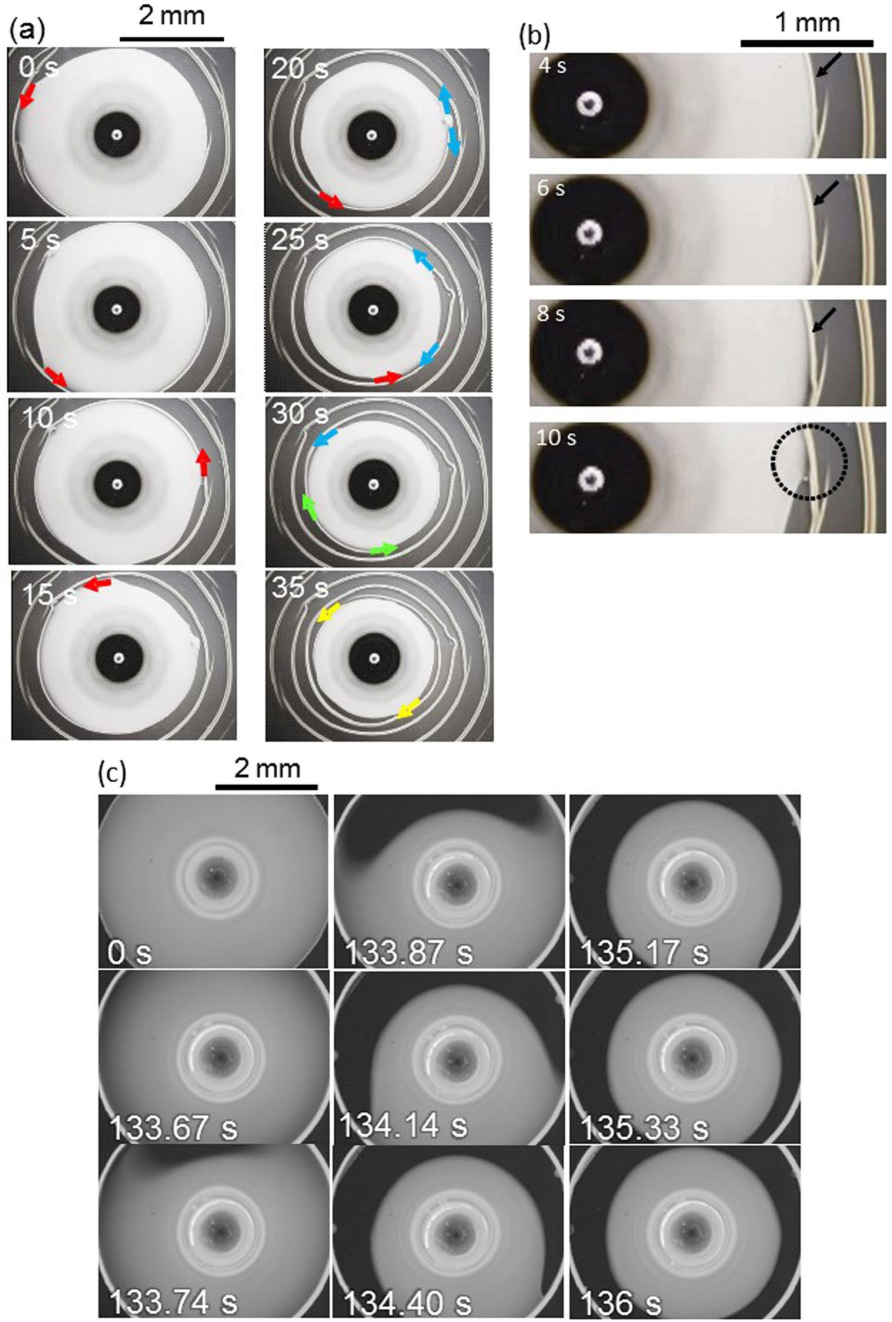
Time course of pattern formation from a droplet containing both SWCNTs and PFO. (**a**) long-term behavior and (**b**) magnifications of images obtained for spiral pattern formation between 4 and 10 s. A glass plate is used as the solid surface. (**c**) Fluorescence from PFO is observed for concentric-ring pattern formation. Glass coated with silicon is used as the solid surface. Concentrations of PFO and SWCNT are 1.0 g/L and 0.33 g/L, respectively.

Figure 4 shows the time course of pattern formation from a droplet containing CNTs and PFO. (See supporting movie 1) Fig. 4a shows the long-term behavior. The photographs between 4 and 10 s are magnified in Fig. 4b. As evaporation proceeds, a ring-like accumulation of solutes is observed along the periphery of the droplet, as indicated by the black arrows. This accumulation is promoted by the convection caused by evaporation^24^. This ring-like pattern acts as the precursor along which depinning proceeds, as indicated by the dotted circle in the last panel of Fig. 4b. This depinning continues, behaving like a traveling wave, as indicated by the red arrows in Fig. 4a (see Supporting movie 2, showing the peripheral shape of an evaporating droplet from 15–35 s). From 0 to 15 s, a single traveling wave is observed. If this single traveling wave were to continue slowly throughout the evaporation, the pattern would form a complete spiral. In many cases, depinning occurs elsewhere and propagates in opposite directions simultaneously from a single origin, as shown by the blue arrows of 20−25 s and green arrows at 30 s. However, this event does not disrupt spiral formation: The clockwise traveling component is annihilated during collision with the preceding counterclockwise traveling component. This annihilation plays a crucial role in the highly reproducible spiral formation. The annihilation of chemical waves through collision is a specific characteristic of nonlinear waves under far-from-equilibrium conditions, as in the Belousov–Zhabotinsky reaction^29^. However, when impurities aggregate on the surface, the traveling forms the blanch structure. Examples of such blanching are indicated by yellow arrows in Fig. 3c and d.

When the surface is completely clean and smooth and the traveling speed is high, a concentric-ring pattern forms. Figure 4c shows the result under these conditions, in which fluorescence from PFO is observed. In this experiment, CNTs are also present in the sample; the brightness represents the concentration of PFO. The fluorescence from the periphery increases from 0 s to 133 s, demonstrating that PFO (and CNTs) accumulates at the periphery. After 133 s, a complete ring is formed by depinning, which travels very quickly over a long distance (133.74–140 s). When this event succeeds during evaporation, the concentric-ring pattern is formed (see Supporting movie 3). In our experiments, the appearance of a concentric-ring pattern is noted in only a few cases among hundreds of experimental runs. The low probability of the appearance of the concentric pattern may be attributable to the effect of small aggregates of solute or impurities within the solution. In relation to this, it is well known that concentric-ring patterns are often modified into multiple spirals in the presence of local disturbances, such as small dust particles, in the Belousov–Zhabotinsky reaction^30^.

**Figure 5:**
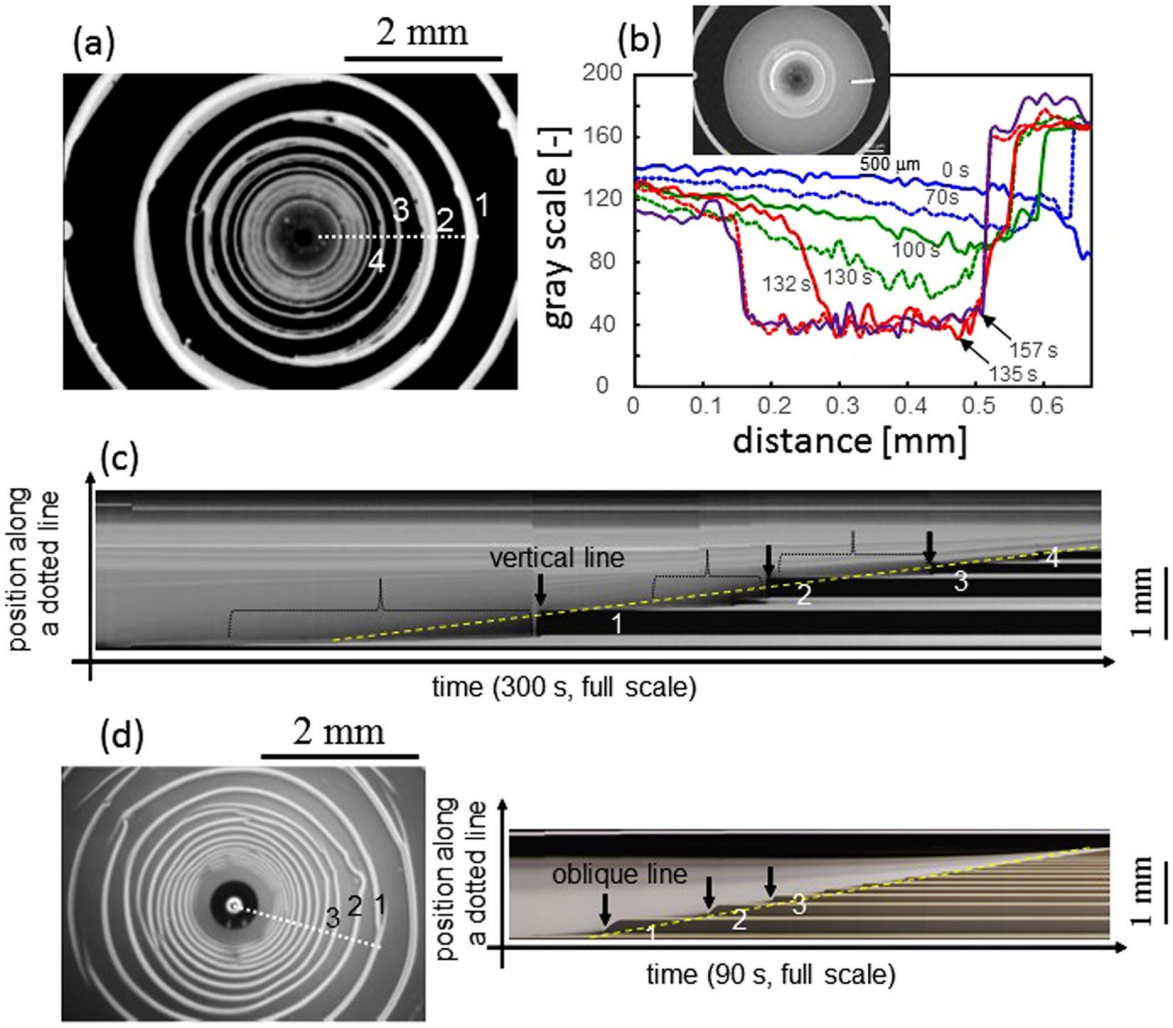
Dynamics of pattern formation. (**a**) Final fluorescence image of concentric-ring pattern. Glass coated by silicon is used as the solid surface. (**b**) Time-course of fluorescence intensity (gray scale of the photograph) along the line shown in the inset. A glass plate is used as the solid surface. (**c**) and (**d**) show, respectively, a space-time plot of pattern formation along the line shown in (**a**) and in the left image in (**d**). For both experiments, the concentrations of PFO and SWCNT are 1.0 g/L and 0.33 g/L, respectively.

Figure 5a and c, respectively, show the fluorescence intensity of the final concentric-ring pattern and a space-time plot along the dotted line of Fig. 5a. The space-time plot indicates that stripes form after very quick traveling of depinning (vertical lines indicated by black arrows). In contrast, depinning occurs more slowly in the spiral pattern, as shown in Fig. 5d, in which a stripe forms after an oblique line in the space-time plot (oblique lines indicated by black arrows). Note that Fig. 5d is not a fluorescence image, but instead a bright-field micrograph. In both patterns, the speed of contact-line retreat is independent of time (Fig. 5c and d show that an envelope of the white region is correlated with a yellow dashed line). In Fig. 5c, fluorescence intensity at the positions indicated by dotted braces decreases with time, while that at the stripe increases simultaneously. This is shown quantitatively in Fig. 5b. This result demonstrates that solute molecules near the stripe accumulate along the stripe, as mentioned in the discussion of Fig. 2b. This accumulation is probably caused by evaporation-induced convection. The accumulation of solutes is also observed for the spiral pattern shown in Fig. 5d (black arrow).

### Alignment of CNTs

**Figure 6:**
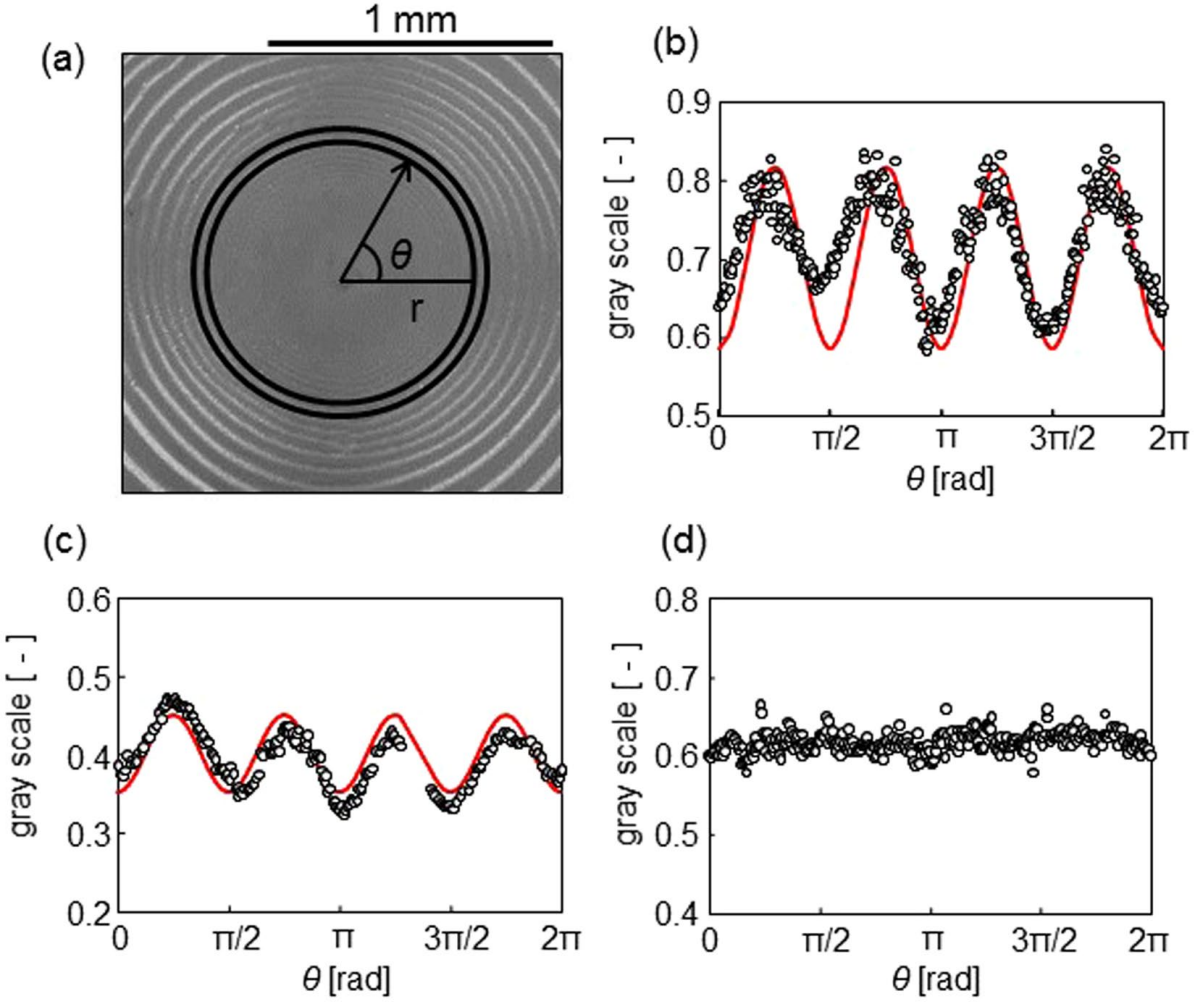
Polarized optical microscopy image of a drying pattern containing SWCNTs and PFO. (**a**) The micrograph on a glass surface. (**b**) and (**c**) show the gray scale as a function of the azimuth angle for the glass surface and silicon wafer, respectively. (**d**) Gray scale of optical microscopy image of PFO alone. The concentrations of PFO and SWCNT are 1.0 g/L and 0.33 g/L, respectively. For experiment of (**d**), SWCNT was not contained. A glass plate is used as the solid surface.

Figure 6 shows a polarized optical micrograph of the spiral pattern in a cross-Nicol alignment. Optical anisotropy is clearly observed in the image. Brightness within the ring shown in Fig. 6a is expressed in gray scale (0−1), and the dependency on the azimuth *θ* (see Fig. 6a) is shown in Fig. 6b for the glass surface and Fig. 6c for the silicon wafer. When the pattern is optically anisotropic and the principal axes of the refractive indices are parallel to the stripe and its orthogonal direction, the intensity of transparent light is proportional to sin^2^(2*θ*), shown as a solid curve in Fig. 6b and c (the actual result is expressed as *a*sin^2^(2*θ*) + *b*, where *a* and *b* are constants). The experimental results are explained by this curve. Optical anisotropy is not observed for the spiral patterns formed by PFO alone (Fig. 6d). This suggests that the optical anisotropy shown in Fig. 6a–c is caused by the alignment of SWCNTs. The optical anisotropy caused by SWCNT is observed for different stripes in a pattern. The result is shown in Figure S4, indicating that the anisotropy of an inner stripe appears to be a little weaker than that of outer one. (See Supporting Figure S4)

We have also performed the measurement with Raman polarization microscopy in order to evaluate the alignment in a quantitative manner^31^. Unfortunately, we have failed to obtain favorable spectra with enough contrast, which is attributable to the presence of relatively large amount of PFO in our samples. Future re-trial will provide important information on the alignment of CNTs.

**Figure 7:**
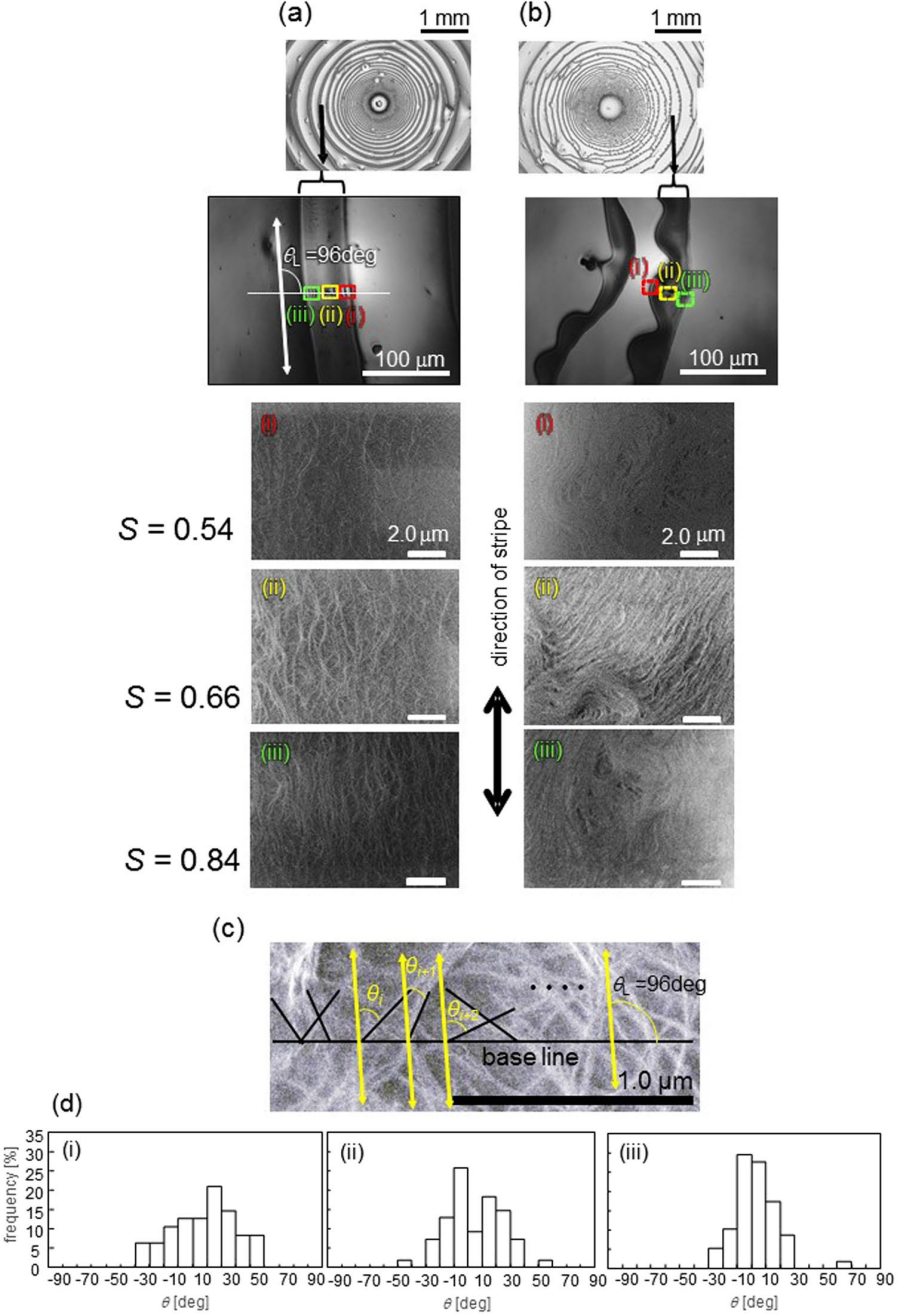
Scanning electron microscopy images of (**a**) smooth and (**b**) wavy patterns with quantification of ordering in SWCNT alignment (**c**,**d**). (**a**,**b**) The top two panels of each column are obtained by laser microscopy. The direction of the stripe is equal in the SEM and laser microscopy images. (**c**) Definition of *θ*_i_ in eq. 1. The base and yellow oblique lines, respectively, are the same as those shown in (**a**). (**d**) Distribution of the angle calculated as *θ*_i_. The concentrations of PFO and SWCNT are 1.0 g/L and 0.33 g/L, respectively. A silicon wafer is used as the solid surface.

Figure 7 shows the results of laser microscopy (top two images in each column) and scanning electron microscopy (SEM) (remaining three images in each column). This experiment was performed with a silicon wafer substrate because electrical conductivity is required for SEM observation. Two types of SWCNT samples are used. The first is the supernatant of the SWCNT solution after 2 months of storage. The other is the sample just after preparation. The disorder or irregularity of pattern diminishes with an increase in the aging time of sample after preparation. (See Figure S5 in the supporting information.) In the first experiment, coarse aggregates comprising both SWCNTs and PFO are precipitated. This result is shown in Fig. 7a. On the contrary, coarse aggregates remain dispersed in the solution in the latter experiment. They form a wavy pattern, shown in Fig. 7b. SEM images of specific portions of a stripe are also shown. Three places in one stripe are selected, corresponding to the inner (i), middle (ii), and outer (iii) regions. In the regular (non-wavy) stripe (a), the CNTs are aligned along the stripe direction, while no such alignment is observed in the wavy pattern (b).

The mutual alignment of CNTs is quantified by the parameter *S*, defined by^32^

1 $$S=\langle \cos (2{\rm{\Delta }}{\theta }_{i})\rangle .$$

Here, Δ*θ*_i_ = *θ*_i+1_ − *θ*_i_, and *θ*_i_ is defined as shown in Fig. 7c. The yellow oblique line of 96 degrees is parallel to the white line of *θ*_L_ of Fig. 7a. The average, denoted by an angular bracket, represents the average calculated over 50–60 CNT lines across randomly selected base lines. The parameter *S* increases when the adjacent CNTs are more parallel. The average value of *S* is shown in Fig. 7a. The *S* value corresponding to the outer part is higher, indicating greater mutual alignment. The distribution of *θ*_i_ is shown in Fig. 7d. The peak of the distribution is at approximately *θ*_i_ = 0. This demonstrates that the alignment direction is parallel to the stripe. The distribution peaks again at the outermost part of the stripe (iii). Thus, we can conclude that the CNTs are aligned parallel to the stripe and that the CNTs in the outer part in a stripe exhibit higher degrees of ordering.

Recently, Arnold, *et al*., has reported successful parallel alignment of CNTs, by adapting an experimental procedure to deposit CNTs on a solid substrate pulled up gradually, i.e., a kind of Langmuir-Blodgett film^33, 34^. They demonstrated the formation of linear banding of aligned CNTs on the order of ca. 10μm and that such straight bands are arranged in a parallel manner with the distance of ca 100μm, through the procedure of repetitive dropping or pipetting of volatile organic solvent with CNTs onto an aqueous solution layer as the subphase. Moreover, He *et al*. has reported, by the repetitive filtration, the degree of CNTs alignment increasing with the thickness of patterns^31^. In the present experiment, the thickness is spontaneously determined. The degree of alignment order may increase if the thickness could be controlled by experimental procedures. Here, it is noted that we have shown that banding pattern is obtained spontaneously without any artificial repetitive handling procedure, together with the successful construction of circular banding of CNTs which would be the first discovery as far as we aware.

CNTs are rarely dispersed in solvents without dispersants. Thus, the self-patterning of CNTs from liquid phase inevitably accompanies the pattern formation of dispersants. In the present study, the dispersant PFO dominate the pattern formation, causing the alignment of CNTs. Hence, the study of pattern formation dominated by PFO is indispensable to clarify the self-organization of CNTs. In the present system, the pattern did not depend on CNT concentration when it is less than 0.33 g/L, while the pattern was affected by the molecular weight of PFO. (The latter result is not shown.) Various types of CNTs pattern may be realized by selecting the suitable dispersants.

## Discussion

**Figure 8:**
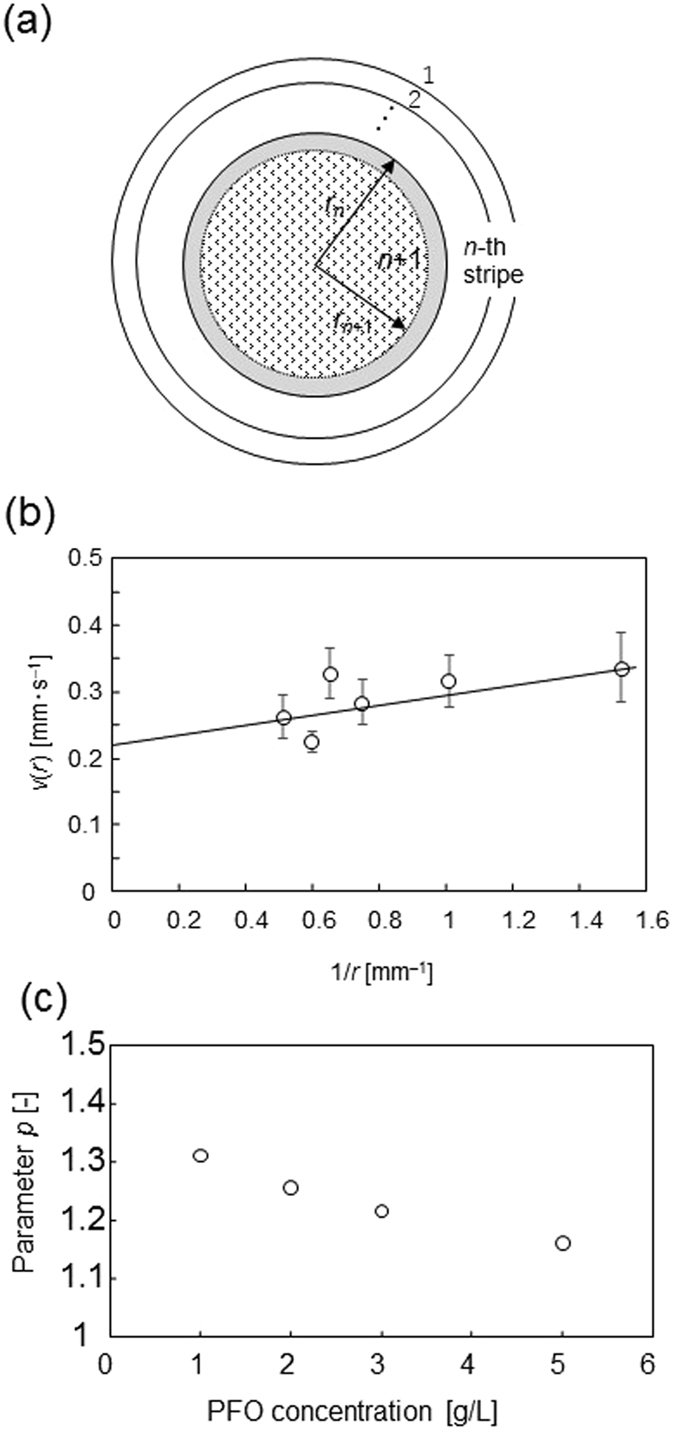
Schematic of pattern formation. (**a**) Illustrated definitions of *r*_n_ and the area of solute depletion. A concentric-ring pattern is drawn, but a spiral may form as well. The inner area of the *n*-th stripe is inside the droplet. The *n*-th stripe is forming, and the position where the (*n* + 1)-th stripe will be formed is also shown. (**b**) Speed of the traveling wave as a function of 1/*r* for the experiment shown in Fig. 4a. (**c**) The enrichment parameter *p* is shown as a function of PFO concentration.

The spiral pattern is formed by traveling waves, as shown in Fig. 4a. The velocity of a traveling wave along the circular periphery of the *n*-th stripe (Fig. 8a) (*v*_n_) is given by *v*_n_ ≈ 2π*r*_n_/*t*_n_, where *t*_n_ is the time required for the formation of the *n*-th stripe. Figure 5c and d indicate that the speed of contact line retreat (*v*_r_) is approximately constant during pattern formation for both spirals and concentric rings. *v*_r_ is expressed by *v*_r_ = (*r*_n_−*r*_n+1_)/*t*_n_. This yields *r*_n+1_ = *r*_n_(1−2π*v*_r_/*v*_n_). Figure 3f shows that *r*_n_ follows a geometric sequence, *r*_n+1_/*r*_n_ = exp(−*β*). This results in exp(−*β*) = 1−2π*v*_r_/*v*_n_. Since *v*_r_ is approximately constant (Fig. 5c and d), *v*_n_ is independent of *n* (or *r*_n_) at a fixed PFO concentration.

Circular geometries forming spirals or concentric rings are often formed in nonlinear pattern formation of the reaction-diffusion type. Then, the velocity of the traveling wave follows *v*(*r*) = *v*_0_ + *D*/*r* ^35^. Here, *v*_0_ and *D* denote the velocity at zero curvature and the diffusion constant. Figure 8b shows *v*(*r*) for the experiment shown in Fig. 4 as a function of 1/*r*. Here, *v*_n_ and *n* at corresponding *r* are plotted. The dependency on *r* is weak. In the present system, *v*_0_ dominates the traveling speed: The diffusion term (mass transport driven by the concentration gradient) is not significant. This suggests that the traveling wave is mainly controlled by hydrodynamic properties. *v*_r_ is determined by the evaporation rate at the edge of the droplet. This is nearly constant and independent of *r* (Fig. 5d). Both *v*_r_ and *v*_n_ are nearly independent of *n* (or *r*), resulting in the geometric sequence for *r*_n_ shown in Fig. 3f.

Figure 3e and g indicate that *w*_n_ is proportional to the PFO concentration at a fixed *n*. Thus, the result of *w*_n+1_/*w*_n_ = exp(−*α*) (Fig. 3e) means that *C*_n+1_/*C*_n_ = exp(−*α*), where *C*_n_ is the solute concentration in a droplet while forming the *n*-th stripe (see Fig. 8a; stripes from 1 to *n*−1 have already formed). The decrease in width with increases in stripe number (Fig. 3e) may be caused by decreases in the PFO concentration during pattern formation, i.e., *C*_n+1_ < *C*_n_. This is possible only when the solute molecules are enriched at the droplet periphery: The enriched solutes are deposited at the periphery by solvent evaporation, which reduces the PFO concentration in a droplet. This is indicated by the observation shown in Fig. 5b: The fluorescence intensity of a stripe increases as it forms, accompanied by the reduction of fluorescence intensity in the region of the next stripe.

Consider the *n*-th stripe. In formation, this stripe absorbs the solute molecules in the gray-colored area range shown in Fig. 8a. Here, we may assume that the (*n* + 1)-th stripe will be formed at *r*_n+1_, shown in Fig. 8a. This assumption is supported by the result of Fig. 5c, in which the *n*-th stripe appears at the edge of the less fluorescent zone. The mass conservation of solute molecules is expressed by

2 $$\pi {r}_{n}^{2}{C}_{n}=\pi {r}_{n+1}^{2}{C}_{n+1}+\pi ({r}_{n}^{2}-{r}_{n+1}^{2})p{C}_{n}$$

Here, we assume that the thickness of the droplet remains constant and that the solute molecules are enriched to *pC*_n_ (*p* > 1) in the gray-colored area. Considering *r*_n+1_ = exp(*−β*)*r*_n_ into accou_n_t, we obtain

3 $$\frac{{C}_{n+1}}{{C}_{n}}={e}^{2\beta }-({e}^{2\beta }-1)p$$

Using *C*_n+1_/*C*_n_ = exp(−*α*), we obtain

4 $$p=1+\frac{1-{e}^{-\alpha }}{{e}^{2\beta }-1}$$

The second term of the right-hand side corresponds to the degree of solute enrichment at the periphery. Using *α* ~ 0.06 and *β* ~ 0.017[PFO](g/L) + 0.069, we obtain the value of *p* from eq. 4. The result is shown in Fig. 8c. The solute is more enriched at lower PFO concentrations. This enrichment creates the geometric sequence of *w*_n_.

The geometric sequences for *w*_n_ and *r*_n_ may suggest an analogy to the Liesegang phenomenon for a precipitate in gel^36^. In studies of the Liesegang phenomenon, the rules for the width and position of the precipitate band are discussed based on the reaction-diffusion mechanism. This is because the experiments are performed in gel, with strongly restricted convection. On the contrary, in the present system the role of diffusion is restricted for fluidity in the droplet. These geometric sequences may be based on a more general mechanism, irrespective of the mass transport processes.

Solute enrichment may be enhanced by the alignment of SWCNTs. When rod-like DNA molecules are dispersed in a solution containing globular polymers, such as polyethylene glycol (PEG), the DNA molecules tend to attract each other by the depletion or repulsive effects from the crowded PEG molecules. This causes DNA enrichment near the interface and mutual alignment of DNA molecules^37^. The same mechanism may operate in the present system. This may explain the higher degree of alignment at the outer part of each stripe.

## Methods

SWCNTs were purchased from Unidym (HiPco, 0.8–1.2 nm in diameter and 100–1000 nm in length). The requisite amount of SWCNTs (0.33 g/L) was dispersed by sonication in toluene containing 1.0, 2.0, 3.0, and 5.0 g/L poly(9,9-dioctyl-fluorene-2,7-diyl) (PFO)^28^. PFO was purchased from Aldrich and its average molecular weight was approximately 65,000. After centrifugation at 10,000 *g* for 1 h, the supernatant liquid was used as the sample in the subsequent drying experiments. The supernatant of the SWCNT solution, at least, after 1 month of storage was used to obtain reproducible results. As a dispersant, PFO is known to extract only semiconducting SWCNTs from mixtures of semiconducting and metallic SWCNTs^38^. Figure 1a,b illustrates the drying method^14, 18^. A drop of the SWCNT solution is placed around a glass bead (Fig. 1a) on a glass plate or between the top of a glass bead and plate (Fig. 1b) and dried in either atmosphere or vacuum at room temperature until the solvent is completely evaporated. After evaporation, a mixture of the SWCNTs and PFO remains to form a pattern, which is observed by optical (Olympus, DP71), scanning electron (JEOL, JSM-7500F), and laser (Keyence, VK-X210) microscopies. As solid substrates, plates comprising borosilicate glass or silicon wafers were used. The formed pattern does not strongly depend on the substrate material. (However, patterns drastically changed when hydrophobic substrate (polyvinyl chloride) was used. The effect of substrate surface is exemplified in Supporting Figure S6.) The pattern formation dynamics is monitored by a CCD camera (Keyence, VW-6000/5000) in both bright and dark fields, using the fluorescence of PFO. For the latter case, a UV lamp (As One, SLUV-4 254/365) is used for excitation, and the visible emission is monitored.

## Electronic supplementary material

Supporting Information

supporting movie 1

supporting movie 2

supporting movie 3
