# Supplementary material for: Self-Organized Micro-Spiral of Single-Walled Carbon Nanotubes
Source: Sci Rep. 2017 Jul 13;7:5267. doi: 10.1038/s41598-017-05558-9 (PMC5509688; doi:10.1038/s41598-017-05558-9)
Supplement: Supplementary file 1 — Supporting Information [file 41598_2017_5558_MOESM1_ESM.pdf]

Self-Organized Micro-Spiral of Single-Walled Carbon Nanotubes

Keisuke Mae[1], Hidetoshi Toyama[1], Erika Nawa-Okita[2], Daigo Yamamoto[1], Yong-Jun Chen[3], Kenichi Yoshikawa[4], Fumiyuki Toshimitsu[5], Naotoshi Nakashima[6], Kazunari Matsuda[7], Akihisa Shioi*[1]

[1] Department of Chemical Engineering & Materials Science, Doshisha University, Kyoto 610-0321, Japan

[2] Organization for Research Initiatives and Development, Department of Chemical Engineering & Materials Science, Doshisha University, Kyoto 610-0321, Japan

[3] Department of Physics, Shaoxing University, Shaoxing, Zhejiang Province 31200, China

[4] Faculty of Life and Medical Sciences, Doshisha University, Kyoto 610-0394, Japan

[5] Department of Applied Chemistry, Kyushu University, Fukuoka 819-0395, Japan,

[6] International Institute for Carbon-Neutral Energy Research, Kyushu University, Fukuoka 819-0395, Japan

[7] Institute of Advanced Energy, Kyoto University, Uji, Kyoto 611-0011, Japan

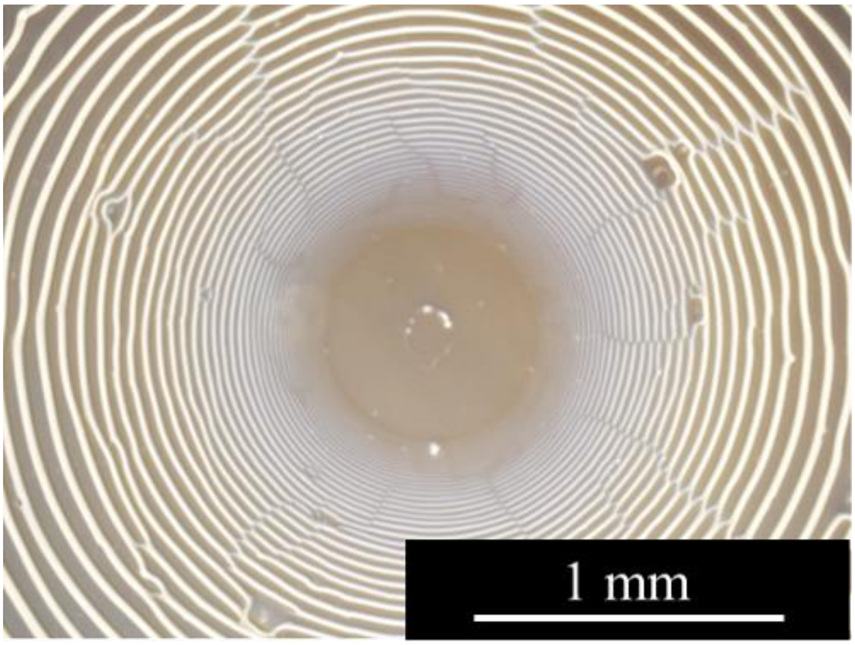

Fig. S1

Patten formed by method 1. The sample contains PFO only. The concentration is 1.0 g/L. Glass plate is used.

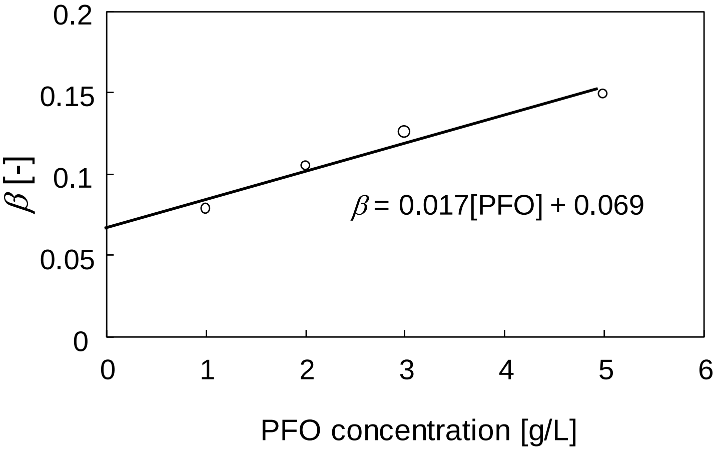

Fig. S2

The exponent β is shown against PFO concentration.

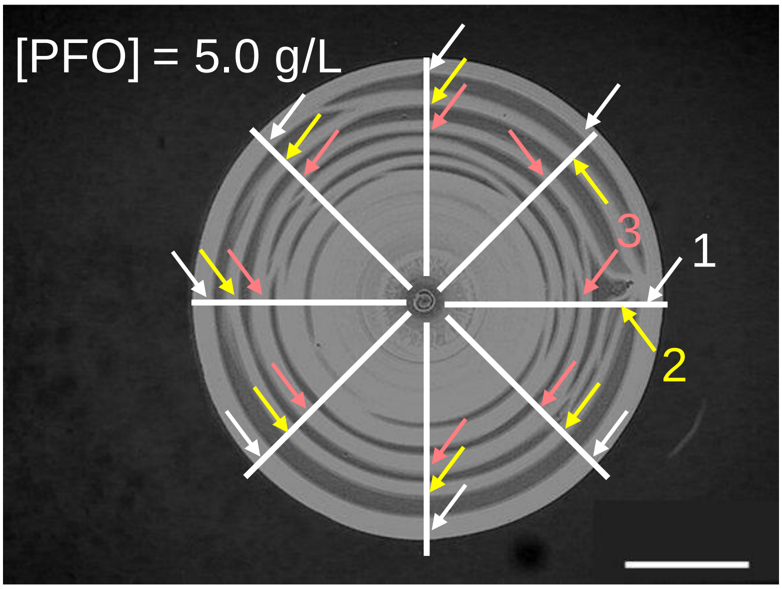

Fig. S3

How to select each stripe in a pattern. Stripe number is given as shown above. The width and position are measured at positions indicated. The results is identical to that of Fig.3d.

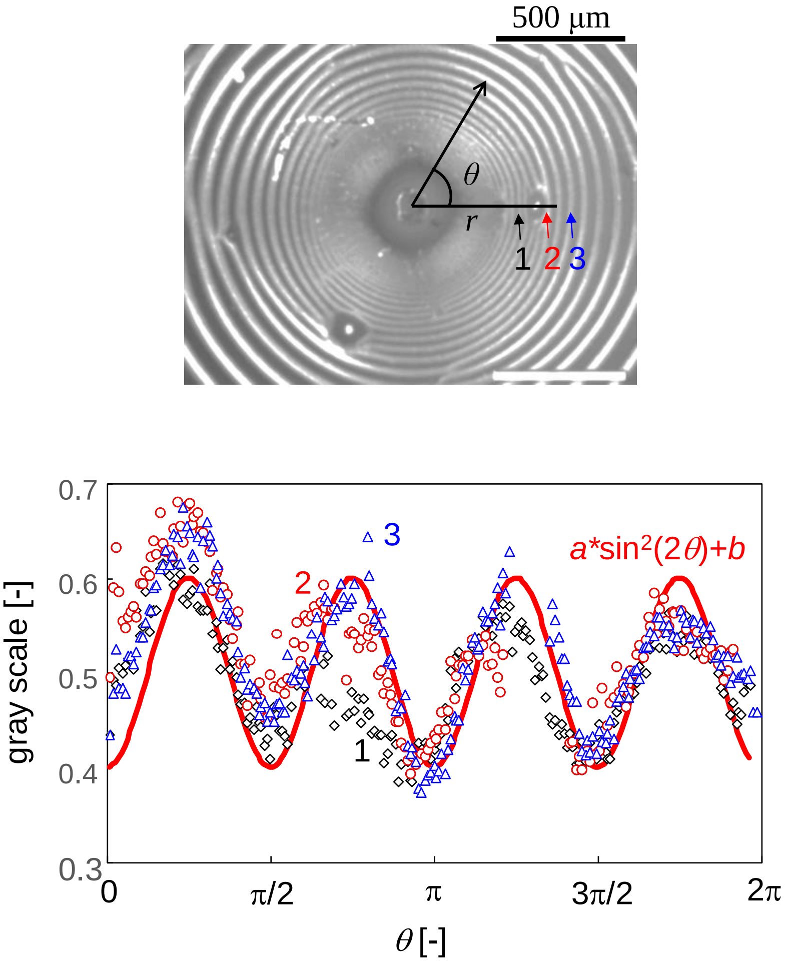

Fig. S4

Optical anisotropy of three stripes in a pattern. The stripes at inner (1), middle (2) and outer (3) positions are shown. The sample is identical to Fig.6c.

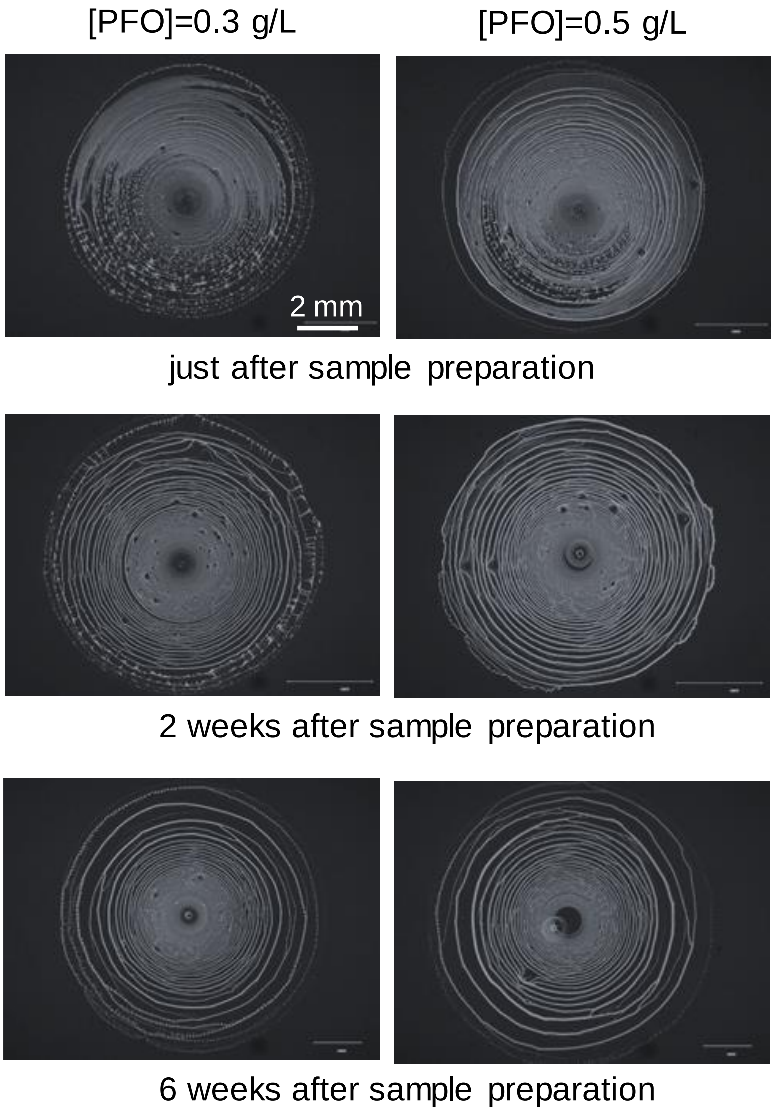

Fig. S5

Effect of aging time of sample. PFO concentration of the left and the right column are 0.3 and 0.5 g/L, respectively. Aging time is shown. Patten is formed by method 1.

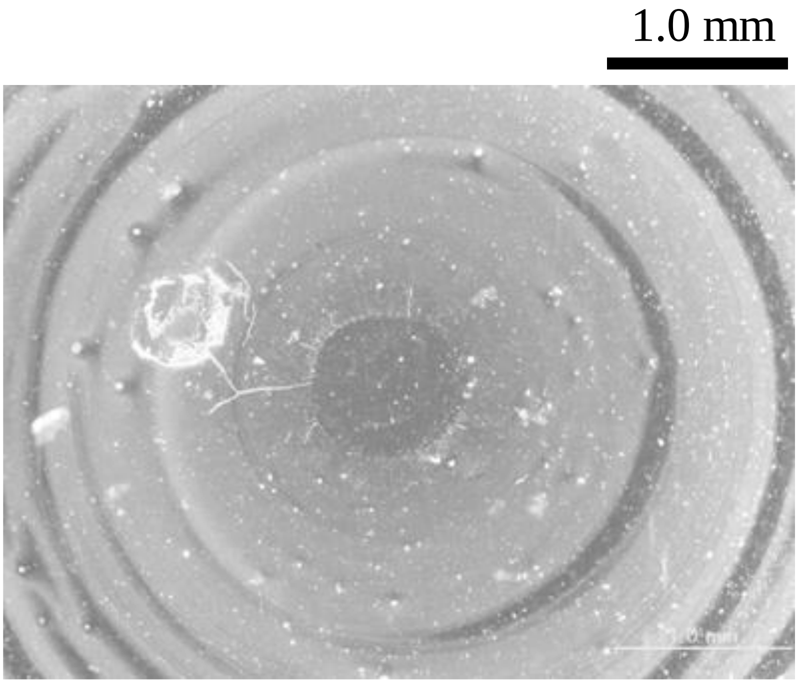

Fig. S6

Drying pattern of PFO on PVC surface for method I. Concentration of PFO is 2.0 g/L. No stripe pattern formed. Instead, the glass surface was covered by PFO layer.
